# Post-intensive care syndrome in primary care: The development of new diseases and primary care services utilisation – a prospective cohort study

**DOI:** 10.1080/13814788.2023.2213476

**Published:** 2023-05-30

**Authors:** Dries van Sleeuwen, Sabine de Man, Marieke Zegers, Reinier Akkermans, Michael Ricking, Marco Peters, Mark van den Boogaard, Floris A. van de Laar

**Affiliations:** a Department of Primary and Community Care, Radboud University Medical Center, Radboud Institute for Health Science, Nijmegen, the Netherlands; b Department of Intensive Care, Radboud University Medical Center, Radboud Institute for Health Science, Nijmegen, the Netherlands; c Radboud University Medical Center, Radboud Institute for Health Sciences, Scientific Institute for Quality of Care, Nijmegen, the Netherlands; d Department of Intensive Care, Canisius Wilhelmina Ziekenhuis, Nijmegen, the Netherlands

**Keywords:** General practice, primary care, intensive care unit, post-intensive care syndrome, critical illness

## Abstract

**Background:**

Patients experience long-lasting health problems defined as post-intensive care syndrome (PICS) after Intensive Care Unit (ICU) admission. Little is known about PICS in primary care.

**Objectives:**

To investigate whether ICU survivors encounter more new International Classification of Primary Care-2 (ICPC-2) diagnoses and general practitioner (GP) contact compared to patients with similar comorbidity without ICU admission.

**Methods:**

Prospective multicentre cohort study in three Dutch general practices. Numbers of disease-episodes and GP contacts of ICU survivors ≥ 16 years admitted between 2008 and 2017 were extracted from GPs’ information systems. A non-ICU reference cohort was matched 1:1 for age, sex, follow-up period and comorbidity groups from patients’ medical history. Negative binominal regression analysis was used to compare both cohorts 0–3, 3–6, 6–12 months, 1–2 and 2–5 years after ICU admission and 1 year prior to admission.

**Results:**

ICU survivors (*n* = 199) encountered more new disease-episodes 1 year before (mean 3.97 (95% confidence interval [CI] 3.50–4.52]]; reference 2.36 [1.28–3.17]) to 2–5 years after ICU admission (3.65 [3.15–4.26]; reference 2.86 [2.52–3.22]). ICU survivors also had more GP contacts 1 year before (mean 19.61 [17.31–22.17]; reference 10.02 [7.81–12.38]) to 2–5 years after ICU admission (18.53 [15.58–21.85]; reference 12.03 [10.33–13.91]). Patients with prior ICU admission did not encounter patterns in specific ICPC-2 chapters compared to non-ICU patients.

**Conclusion:**

Patients admitted to the ICU encounter more new primary care disease-episodes and GP contacts. As patients present their symptoms to their GP first, it is therefore up to the GP to recognise these critical illness-related symptoms.

KEY MESSAGES

- Former ICU patients encounter more new disease-episodes and GP contacts than the comparison group before and 5 years after ICU admission.
- GPs should be alert to ICU-related health problems years after ICU admission.
- Collaboration among primary care, hospitals and community services should be strengthened to mitigate post-ICU health problems.

## Introduction

Many Intensive Care Unit (ICU) survivors experience long-lasting health problems, which is defined as Post Intensive Care Syndrome (PICS) [1–4]. Problems in physical (e.g. fatigue, weakness), mental (e.g. anxiety, depression) and/or cognitive (e.g. loss of memory) health are reported by 50–70% of the ICU survivors due to critical illness and ICU admission [5]. Co-occurrence of these domains is also reported, as 1–30% experience symptoms in two or three domains simultaneously [5,6]. Post-ICU health problems can also have a major impact on work and daily functioning, leading to a decreased quality of life (QoL) [7,8]. Many health problems will be presented to primary care first and it is therefore often up to the general practitioner (GP) to recognise the symptoms of post ICU health problems and intervene [9]. Assuming an ICU mortality of 8.7% in the Netherlands in 2017, GPs have to take care of more than four new ICU survivors per 1.000 patients each year [10,11]. Thereby, most medical specialists specialise in one organ system, which might not be sufficient for detecting and treating the wide range of symptoms patients may experience after ICU admission. This may cause ICU survivors consult their GP more often [12]. However, many GPs may not be familiar with the unique needs of these patients [12]. A recent study showed that 57% of the participating GPs were unfamiliar with the terminology ‘PICS’ and ‘PICS-Family’, and only 14% was aware of ICU follow-up care initiatives [13]. Many interventions exist to treat or prevent post-ICU problems [14] and post-ICU care is recommended by international guidelines but not yet evaluated [15]. To improve the process of early detection and prevention of ICU-related health problems in primary care, it is first essential to gain insight into the primary care services utilisation of patients with prior ICU admission as this is not sufficiently described yet [12,16]. This is an important gap in current literature because differentiating symptoms of post-ICU health problems from those present before ICU admission can be difficult [7]. To do so, it is essential to know whether its presentation differs from other chronic comorbidities. Identifying symptoms of post-ICU health problems more accurately in primary care may lead to more targeted treatment planning and an increased QoL [7,8,17]. Therefore, this study aimed to investigate the difference in new disease-episodes and contact frequency between ICU survivors and matched reference patients without ICU admission.

## Methods

### Design

A prospective cohort study was conducted in three general practices in Nijmegen, the Netherlands. A post-ICU cohort was set up for patients admitted to the ICU between 2008 and 2017 in one of the two major hospitals in Nijmegen (Radboud University Medical Centre and Canisius Wilhelmina Ziekenhuis). Patients without ICU admission were assigned to the reference cohort.

### Study population

Patients were included if they were ≥16 years, admitted to the ICU for one or more days between 2008 and 2017, were registered with one of the three participating general practices by the time of ICU admission and had to survive for at least two days after hospital discharge. Patients were excluded if they had a missing HIS number (general practice software application for registration of patient data), missing or incorrect admission or discharge dates, if they were admitted to the other hospital first or when they had a follow-up time of one day or less. Each patient with prior ICU admission was matched to one reference patient [18], registered in the same period in one of the participating general practices. Reference patients were matched 1:1 based on sex, age (with a range of + or − 5 years), follow-up period and comorbidity. Each patient in the reference cohort was unique. Comorbidities (Supplementary Table S1) were based on the prevalence of diseases in adult patients in primary care [19]. When included patients left the GP practice, e.g. when they switched GP practice, they were marked as a loss to follow up.

### Data collection

Data were collected from the Family Medicine Network (FaMe-Net), a primary care research network subject to systematic quality tests [20,21]. Nine general practices in the Netherlands are part of the network and prospectively and structurally register diagnoses according to the International Classification of Primary Care-2 (ICPC-2) coding system [22]. This study used data from three general practices close to the two participating ICUs. Data were collected 1 year prior to the ICU admission date. The follow-up period had a minimum of 2 days after ICU discharge and a maximum of 5 years. If patients dropped out, data were included until then. Reference patient’s follow-up time was set to meet the exact end dates as their matched patient with prior ICU admission.

### Outcomes

To address differences in presentation between patients admitted to the ICU and patients without ICU admission, the following outcomes were assessed:

1. Quantity and course of developing new disease-episodes, measured with ICPC-2 registration, as reported within respectively −12–0 months before ICU admission (baseline), 0–3 months, 3–6 months, 6–12 months, 1–2 years and 2–5 years after ICU discharge.
2. Frequency and course of GP contact, measured with HIS registrations of GP consults at the general practice or the patient’s home. GP consults by telephone or online consultations were also included.
3. Quantity of registered new disease-episodes as registered per ICPC-2 chapter [23].

### Statistics

Patient characteristics were described with descriptive statistics. Descriptive statistics were also used to calculate the quantity of reported new ICPC-2 registrations and GP contact frequencies for the patients admitted to the ICU and the reference patients using means with 95% confidence interval (CI). Negative binomial regression analysis was used to express a Rate Ratio (RR) and Rate of two Rate Ratios (RRR) for the differences in new disease-episodes, contact frequency and new disease-episodes per ICPC-2 chapter [24]. A RR represents a relative equation of the mean counts per year in a specified timeframe compared to the mean counts per year at baseline for both patients with prior ICU admission and patients without and is not equivalent to a risk difference or a rate difference. The course of new disease-episodes and contact frequency was analysed with the RRR, which is an equation of the two relative changes of mean counts for the patients with prior ICU admission compared to those without. Registered new ICPC-2 chapters were analysed exploratively and therefore no correcting for multiple testing was applied. Analyses were performed for the total study period, starting the year before admission and a maximum of 5 years after discharge. A *p*-value < 0.05 was considered significant, based on two-sided testing. All analyses were performed using the SPSS statistical package (version 25; IBM).

For this prospective cohort study, the total number of included patients admitted to the ICU was determined by the total number of patients admitted to the ICU during the period in question and matched the inclusion criteria. Subsequently, reference patients were selected and matched. For this reason, power analysis was not applicable.

## Results

### Study population

**Figure 1.**
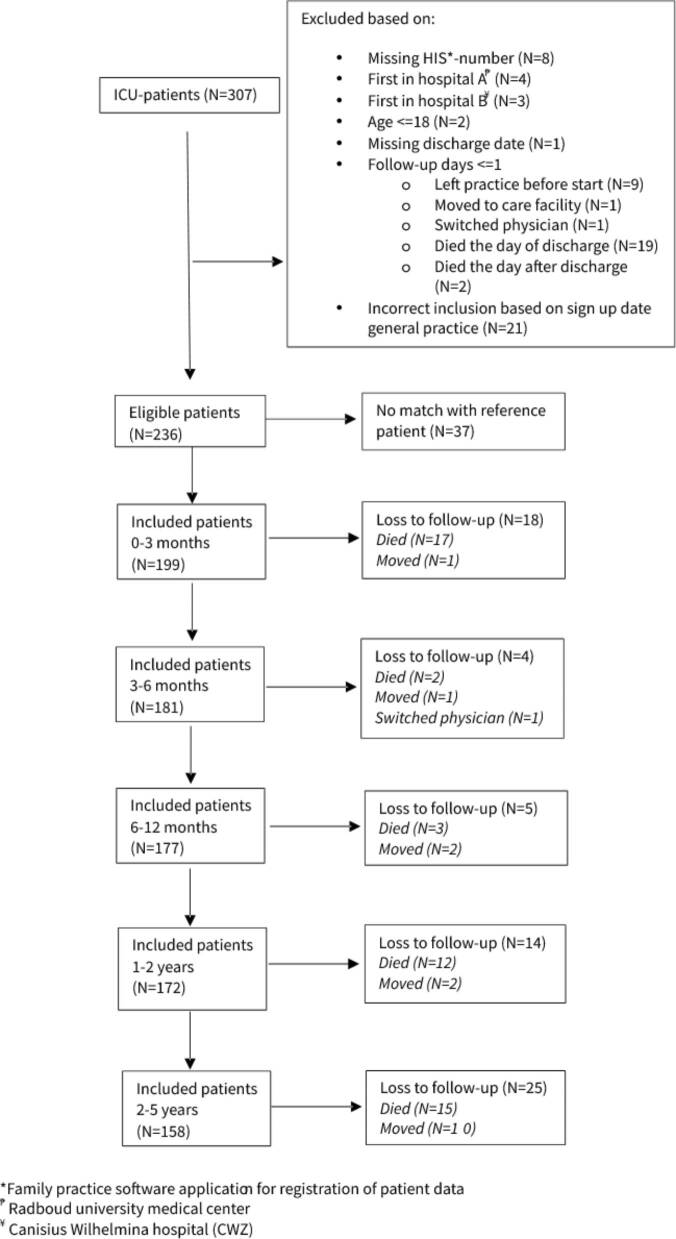
Flowchart of study population.

Between January 2008 and September 2017, 307 patients were admitted to one of the two participating ICUs (Figure 1). Of these patients, 236 were eligible for inclusion and 199 could be matched with a reference patient taking the matching criteria into account. In total, 49 patients died during the 5-year follow-up period. ICU-specific data was registered for 184 patients (Table 1). Most patients admitted to the ICU had an elective surgical reason for admission (54.9%). The median age of both cohorts was 63 year and the majority was male (66.7%). Reference patients were matched for comorbidity groups, however, distribution of comorbidity sub-groups differed slightly (Supplementary Table S1). Diabetes, COPD and immunological insufficiency were the most frequent comorbidities in patients admitted to the ICU.

**Table 1.** Demographic and clinical characteristics of patients in the former ICU and reference cohort presented in median (25th–75th percentile) or *N*(%).

	Former ICU cohort (*N* = 199)	Reference cohort (*N* = 199)
Sex (female/male)	66 (33.2)/133 (66.8)	66 (33.2)/133 (66.8)
Age (mean [*SD*])	60.7 [15.5]	60.6 [15.5]
ICU hospital (Radboudumc/CWZ)	125 (62.8)/74 (37.2)	
ICU variables	*N* = 184*	
Length of ICU stay (days)	2.0 (2.0 − 3.0)	
Comorbidity at ICU admission		
Diabetes	26 (10.2)	
COPD	17 (6.7)	
Immunological insufficiency	17 (6.7)	
Dysrhythmia	15 (5.9)	
CPR	10 (3.9)	
Neoplasm	7 (2.7)	
Chronic renal insufficiency	6 (2.4)	
Stroke	6 (2.4)	
Intracranial mass	6 (2.4)	
Haematological malignancy	5 (2.0)	
Chronic cardiovascular insufficiency	5 (2.0)	
Chronic respiratory insufficiency	4 (1.6)	
Liver cirrhosis	2 (0.8)	
Gastro-intestinal bleeding	1 (0.4)	
APACHE-II score (mean [*SD*]) (*N* = 180)	14.8 [6.1]	
Mechanical ventilation (days) (*N* = 183)	1 (0-2)	
Admission type		
Medical	59 (32.1)	
Emergency surgery	24 (13.1)	
Planned surgery	101 (54.9)	
Length of hospital stay (days)	8.0 (5.0 − 14.0)	

Abbreviation: APACHE: Acute Physiology and Chronic Health Evaluation; ICU: Intensive Care Unit; IQR: Interquartile range; COPD: chronic obstructive pulmonary disease; CPR: cardiopulmonary resuscitation; CWZ: Canisius Wilhelmina Hospital; *SD*: standard deviation.

*Missing data of patients (*N* = 15).

### New disease-episodes

**Figure 2.**
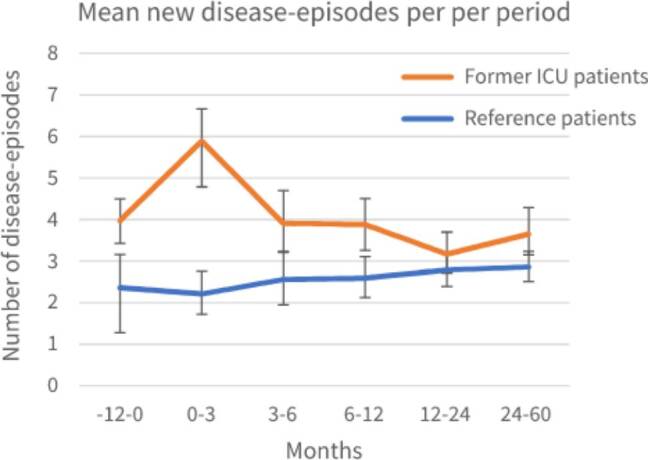
Mean new disease-episodes calculated as mean new disease-episodes per year for each period, for patients with prior ICU admission and reference patients with 95% Confidence Interval (CI).

Overall, the ICU cohort had more new disease-episodes than the reference cohort. One year before ICU admission, patients admitted to the ICU had a mean of 3.97 new disease-episodes per year (95%CI: 3.50–4.52) compared to a mean of 2.36 for reference patients (95%CI: 1.28–3.17). For the first three months after ICU discharge, the mean new disease-episodes for patients admitted to the ICU was 5.89 per year (95%CI: 4.77–7.25), compared to a mean of 2.21 for reference patients (95%CI: 1.71–2.72). 2–5 years after ICU admission, the mean new disease-episodes for patients admitted to the ICU was 3.65 per year (95%CI: 3.15–4.26), compared to a mean of 2.86 for reference patients (95%CI: 2.52–3.22) (Figure 2). Exact mean counts of new disease-episodes per year per period, including a calculation example is shown in Supplementary Table S2.

Calculation examples of RR and RRR are shown in Table 2. The RR of developing a new disease-episode 1 year before ICU admission was 1.55 (95%CI: 1.24–1.95; *p* < 0.05), indicating that before ICU admission, patients admitted to the ICU had 55% more new disease-episodes than reference patients with matched chronic comorbidity without ICU admission. The RR of the ICU cohort at 0–3 months was 1.48 (95%CI: 1.19–1.83; *p* < 0.05) and at 1–2 years 0.81 (0.66–0.99; *p* < 0.05), indicating the number of new disease-episodes was 48% higher and 19% lower respectively when compared to 1 year before ICU admission. For the other timeframes this was not significant. The RRR of developing a new disease-episode at 0–3 months after ICU discharge was 1.59 (95%CI: 1.13–2.25; *p* < 0.05) (Table 2). This indicates that, at 0–3 months after ICU discharge, the course of developing new disease-episodes significantly differed from that of the reference cohort and patients admitted to the ICU encountered more new disease-episodes. Although patients admitted to the ICU encountered more new mean disease-episodes in all timeframes, the course of these absolute numbers was not significantly different after 3 months.

**Table 2.** Rate Ratio (RR) (95%CI) of ICU (*N* = 199) and reference (*N* = 199) cohort respectively is calculated from the total number of new disease-episodes and compared with 1 year before ICU admission (amount of episodes pre-ICU).

Disease-episodes	Rate ratio ICU (95%CI)^¥^	Rate ratio reference (95%CI)^£^	Rate ratio ICU/reference^€^	Rate of two rate ratios (95%CI)^$^
−12–0 months			**1.55 (1.24–1.95)**	
0–3 months	**1.48 (1.19–1.83)**	0.93 (0.71–1.22)		**1.59 (1.13–2.25)**
3–6 months	1.03 (0.81–1.30)	1.01 (0.77–1.32)		1.02 (0.71–1.46)
6–12 months	0.94 (0.76–1.17)	0.98 (0.77–1.24)		0.96 (0.70–1.32)
1–2 years	**0.81 (0.66–0.99)**	1.07 (0.86–1.33)		0.75 (0.56–1.01)
2–5 years	0.91 (0.75–1.11)	1.15 (0.93–1.41)		0.80 (0.60–1.05)

Rate of two Rate Ratios (RRR) (95%CI) for a new disease-episode in each post-ICU timeframe compared to reference cohort in comparison with 1 year before ICU admission of the matched patient. Statistically significant difference (*p* < 0.05) in bold.

Calculation example:.

^¥^[calculated mean new disease-episodes per year for ICU patients for specific period]/[calculated mean new disease-episodes per year for ICU patients for period **−**12–0 months].

^£^[calculated mean new disease-episodes per year for reference patients for specific period]/[calculated mean new disease-episodes per year for reference patients for period **−**12–0 months].

^€^[calculated mean new disease-episodes per year for ICU patients for period **−**12–0 months]/[calculated mean new disease-episodes per year for reference patients for period **−**12–0 months].

^$^[Rate Ratio ICU for specific period]/[Rate Ratio reference for specific period].

E.g. 3–6 months: 1.03 [^¥^]/1.01 [^£^] = 1.02.

#### Contact frequency

**Figure 3.**
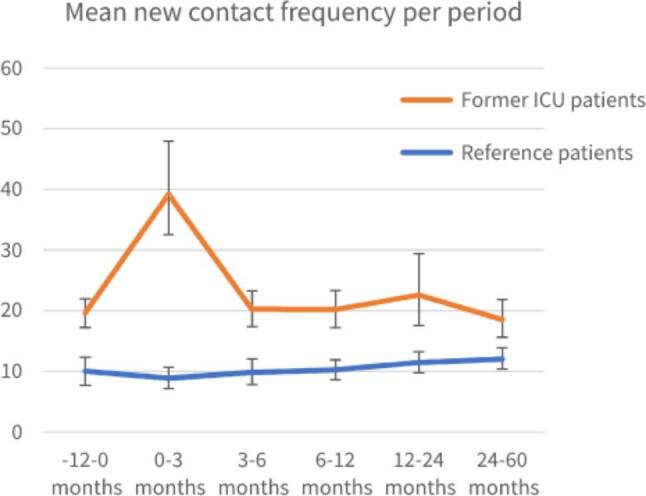
Mean contact frequency calculated as mean contacts per year for each period, for patients with prior ICU admission and reference patients with 95% Confidence Interval (CI).

Patients admitted to the ICU also contacted their GP significant more frequently 1 year before and up to 5 years after ICU admission compared to matched reference patients (−12–0 months: patients admitted to the ICU [mean 19.61, 95%CI: 17.31–22.17]; reference patients [mean 10.02, 95%CI: 7.81–12.38]; 2–5 years: patients admitted to the ICU [mean 18.53, 95%CI: 15.58–21.85]; reference patients [mean 12.03, 95%CI: 10.33–13.91]) (Figure 3). Exact mean counts of new contacts per year per period, including a calculation example is shown in Supplementary Table S3.

The RR for contact frequency in the year prior to ICU admission compared to the reference cohort was 2.19 (95%CI: 1.71–2.80) (Table 3), indicating that the ICU cohort already had over two times more contact with their GP before ICU admission. At 0–3 months after ICU discharge this was 1.87 (95%CI: 1.53–2.28, *p* < 0.05) for the ICU cohort indicating more contact after discharge than before ICU admission. For later timeframes, no significant difference was seen. For the reference cohort, a RR of 1.27 (95%CI: 1.03–1.57, *p* < 0.05) was observed in the 2–5 year period, indicating an increase in contact frequency compared to baseline. A difference in the course of contact frequency was also found in the period of 0–3 months (RRR 2.12; 95%CI: 1.57–2.87; *p* < 0.05) between ICU and reference cohort compared to baseline. For the other timeframes the results were not significantly different (Table 3).

**Table 3.** Rate Ratio of ICU (*N* = 199) and reference (*N* = 199) cohort respectively is calculated from the total number of new contacts and compared with 1 year before ICU admission.

Consultation frequency	Rate ratio ICU	Rate ratio reference	Rate ratio ICU/reference	Rate of two rate ratios (95%CI)
−12–0 months			**2.19 (1.71–2.80)**	
0–3 months	**1.87 (1.53–2.28)**	0.88 (0.70–1.11)		**2.12 (1.57–2.87)**
3–6 months	0.99 (0.80–1.22)	0.91 (0.72–1.15)		1.09 (0.80–1.49)
6–12 months	0.95 (0.77–1.17)	0.96 (0.77–1.20)		0.97 (0.73–1.33)
1–2 years	0.94 (0.77–1.15)	1.15 (0.93–1.42)		0.82 (0.61–1.10)
2–5 years	0.99 (0.80–1.21)	**1.27 (1.03–1.57)**		0.77 (0.58–1.04)

Rate of two Rate Ratios (95%CI) is calculated for contact frequency in each post-ICU timeframe compared to the reference cohort and 1 year before ICU admission. Statistically significant difference (*p* < 0.05) in bold.

#### New disease-episodes clustered by ICPC-2 cohort

New disease-episodes for a specific timeframe were compared to the new disease-episodes 1 year before ICU admission for both the patients admitted to the ICU and matched reference cohort (Supplementary Table S4). For most ICPC-2 chapters, significant increases in new disease-episodes were seen from 3 to 12 months after ICU discharge for both cohorts. For Chapter N (neurological) new disease-episodes, a RR of 5.09 (95%CI: 3.56–7.26, *p* < 0.05) was observed in patients admitted to the ICU in the period of 0–3 months compared to 1 year before ICU admission. After 2 years, a decrease in new disease-episodes was seen compared to 1 year before ICU admission for almost all chapters for both the patients admitted to the ICU and the matched reference cohort.

To explore the course of developing new disease-episodes, little difference was found between patients admitted to the ICU and the reference patients. A significant RRR was only observed in chapter A (general) at 0–3 months (RRR 1.60 [95%CI: 1.06–2.42]) and chapter W (pregnancy, childbearing and family planning) at 1–2 and 2–5 years (RRR 0.42 [95%CI: 0.26–0.69] and 0.49 [95%CI: 0.31–0.77] respectively) (Supplementary Table S5). This indicates that patients admitted to the ICU developed more new disease-episodes in chapter A and fewer in chapter W compared to the reference cohort in this period.

## Discussion

### Main findings

This study showed that patients admitted to the ICU encountered more new disease-episodes and consulted their GP more than matched patients without ICU admission. The course of developing new disease-episodes and GP contact significantly differed from that of the reference cohort at 0–3 months after ICU discharge, whereby patients admitted to the ICU encountered more new disease-episodes (RRR 1.59 [95%CI: 1.13–2.25; *p* < 0.05]) and GP contacts (RRR 2.12; 95%CI: 1.57–2.87; *p* < 0.05).

### Strengths and limitations

A strength of this study is the use of FaMe-Net, guaranteeing unambiguously registered high-quality data of multiple general practices. Long-term follow-up studies on health problems after ICU treatment often are questionnaire studies that primarily focus on patient-reported outcomes. However, questionnaire data can be subject to non-response bias, recall bias and underrepresentation of older or more severely ill patients [6,25,26]. The FaMe-Net database provided prospectively collected data in this study. Another strength is the long-term follow-up time and using pre-ICU admission data. As previous studies often provided a biased picture of ICU survivors by not considering pre-existing comorbidities [27], we created a reference cohort matched for pre-ICU comorbidities, age and sex. Nonetheless, this study has some limitations. As questionnaire research also collects mild problems and measures patients’ quality of life, this information is not collected in administrative databases. Health problems are only included if they were registered as such, and may therefore depend on patients’ help-seeking behaviour and GPs’ coding. Yet, registered diagnoses in this study’s database are assessed by, therefore, adequately trained FaMe-Net GPs. Furthermore, although the matching procedure contained several possible comorbidities, we could not precisely match the severity of these comorbidities. This might have caused a difference in disease severity between the two cohorts. Also, the matching procedure did not consider admission to an ICU in another (not participating) hospital, so reference patients might have been admitted to another ICU, possibly leading to an underestimation of the results. Also, counts of new disease-episodes and contacts could be affected by visits to another GP or hospital specialist. However, in the Netherlands, patients can only be registered with a single GP and need a GP referral for a specialist’s consult. Subsequently, GPs receive specialist’s letters after discharge or consults but only new disease-episodes that require further action of the GP will be registered. When patients visit another GP by exception, such as the General Practice Centre for medical emergencies outside office hours, this will be documented in patients’ medical files likewise. Third, the former ICU patients in this study were admitted to the hospital prior to the COVID-19 pandemic but a small number of patients had their follow-up during this period, which could have influenced the number of consults [28]. However, this applies to both studied cohorts. Finally, as all ICPC-2 chapters and multiple timeframes were taken into account, several analyses were performed but without correcting for multiple testing. However, registered new ICPC-2 chapters were analysed exploratively. Furthermore, the largest group of patients enrolled in the model had an elective type of admission. As those patients generally have better outcomes [5,29], the medical and emergency surgery patients might be underrepresented and therefore the study sample’s results might be slightly better than ICU survivors in general. The percentage of elective surgical patients in the total study population (54.9%) is higher than the national percentages of elective surgical admissions for i.e. the Netherlands (35.1% [2018] and 31.5% [2020]) and Australia and New Zealand (36.6% [2020]) [10,30–32]. In the UK, lower percentages were found (19.2% (2019), 17.7% (2020) and 17.4% (2021)) [33]. However, previous research showed that not only emergency surgical and medical patient are at risk of developing new physical, mental and cognitive health problems due to the period of critical illness but elective surgical patients as well [5].

### Comparison with existing literature

A higher number of new disease-episodes and a higher contact frequency was found among patients admitted to the ICU compared to patients without prior ICU admission. Compared to the reference patients, the highest number of new disease-episodes and contact frequency was found 0-3 months after hospital discharge. This is in line with previous research [12]. After three months, patients admitted to the ICU remain to have significant more GP consultations compared to the matched reference patients. A recent study with similar methods and the same database origin found that relatives of patients admitted to the ICU also present more morbidity in primary care compared to relatives of chronically ill patients after ICU admission, making the health aspects of critical illness far more wide-ranging than the diagnosis of ICU admission itself [34]. Additionally, a remarkable finding was that patients admitted to the ICU also encountered more new disease-episodes and GP contacts 1 year before ICU admission compared to patients without ICU admission. Previous research found that pre-ICU health status was strongly associated with post-ICU health problems and that healthcare costs of ICU survivors are higher compared to patients without ICU admission [5], even the year before ICU admission [35]. This shows that post-ICU health problems are not a static set of symptoms but rather a chronic illness strongly related to pre-ICU functioning and changes through ICU stay and thereafter. Therefore, assessing patients’ pre-ICU health is also important and focussing post-ICU care only on new problems might be too short-sighted [36].

### Implications for research and practice

As patients present their symptoms to their GP first, it is therefore up to the GP to recognise these critical illness-related symptoms. The present study did not show a clear pattern in specific ICPC-2 chapters patients admitted to the ICU might be at risk the most. Still as the phenomenon PICS comprises a prevalent amount of diseases and complaints, it could be difficult and deceptive to be tempted to focus on diagnosing a single complaint. GPs should be extra alert after critical illness and use their holistic approach to diagnose or prevent possible symptoms in all three domains of PICS (physical, mental and cognitive). For this reason, GPs play a pivotal role in post-ICU care, as frequent visits to GPs might lead to early recognition and treatment [37]. Thereby, collaboration after hospital discharge among primary care, hospitals and community services should be strengthened [37,38]. As the COVID-19 pandemic leads to more awareness about long-term consequences of critical illness, post-ICU care is recommended by international guidelines but still not evaluated. Therefore randomised controlled trials involving GPs are needed to evaluate post-ICU care [14,15].

In this study, ICPC-2 chapters were analysed exploratorily. However, the total number of patients per chapter might be too low to conclude differences. Therefore, a larger cohort study might be needed as it would be interesting to know what kind of conditions patients admitted to the ICU might be at risk for. Also, as PICS is a comprehensive syndrome considering three health domains, it might be difficult to frame it using ICPC-2 codes. So, to describe PICS in primary care further, one should think of a solution to this challenge. It could be possible to, e.g. create an ICPC-2 code for PICS in particular, or compare PICS to other (sub)acute onset diagnoses with a chronic cause (e.g. severe trauma, Lyme disease or covid-19). Although the median length of ICU stay of the studied population was 2.0 days (median Dutch length of ICU stay in 2018 was 1.1 days, interquartile range [IQR] 0.8–2.8] [10]), it would finally be interesting to investigate a subgroup analysis for different ICU admission types and different lengths of ICU stay, as outcomes differed in previous research [5].

## Conclusion

This study showed that patients admitted to the ICU developed more new disease-episodes and had more encounters with GPs after discharge compared to non-ICU patients with similar (co)morbidity. Because of possible imperfections in the matching procedure, these results should be confirmed in larger studies.

## Supplementary Material

Supplemental Material
