# Supplementary material for: Post-intensive care syndrome in primary care: The development of new diseases and primary care services utilisation – a prospective cohort study
Source: Eur J Gen Pract. 2023 May 30;29(1):2213476. doi: 10.1080/13814788.2023.2213476 (PMC10231043; doi:10.1080/13814788.2023.2213476)
Supplement: Supplemental Material [file IGEN_A_2213476_SM4370.docx]

Supplementary Table S1. Matching based on pre-existent comorbidities for the ICU (*N* = 199) and reference cohort (*N* = 199).

| Comorbidities | | ICU cohort (N) | Reference cohort (N) |
| --- | --- | --- | --- |
| Neoplasma | | **51** | **51** |
|  | Malignancy | 51 | 51 |
| Cardiovascular disease | | **127** | **127** |
|  | Ischemic heart disease | 62 | 37 |
|  | Heart failure | 15 | 6 |
|  | Cardiac arrythmia | 28 | 20 |
|  | Hypertension | 76 | 82 |
|  | CVA | 0 | 0 |
|  | Cardiovascular other | 57 | 29 |
| Infectious disease | | **0** | **0** |
| Psychological | | **54** | **54** |
|  | Alcohol abuse | 7 | 5 |
|  | Psychiatric illness | 49 | 50 |
|  | Developmental disorder | 4 | 0 |
| Skin disease | | **16** | **16** |
| Asthma/COPD | | **35** | **35** |
| Congenital | | **2** | **2** |
| Congenital abnormalities | | 2 | 2 |
| Other | | **151** | **151** |
|  | Haematological disease | 7 | 2 |
|  | Gastro-intestinal disease | 10 | 11 |
|  | Eye disease | 46 | 44 |
|  | Ear disease | 20 | 26 |
|  | Musculoskeletal disease | 86 | 95 |
|  | Neurological disease | 6 | 3 |
|  | Headache | 11 | 16 |
|  | Endocrine disorder | 11 | 4 |
|  | Fat metabolism disorder | 68 | 69 |
|  | Diabetes | 39 | 32 |
|  | Renal problems | 10 | 0 |

Supplementary Table S2. Mean new disease-episodes calculated per period, calculated as mean new disease-episodes per year for each period, for patients with prior ICU admission and reference patients with 95% Confidence Interval (CI) as shown in Figure 2.

| Period | Former ICU patients (mean) | 95% CI | Reference patients  (mean) | 95% CI |
| --- | --- | --- | --- | --- |
| -12-0 months | **3.97** | **3.50-4.52** | **2.36** | **1.28-3.17** |
| 0-3 months | **5.89** | **4.77-7.25** | **2.21** | **1.71-2.72** |
| 3-6 months | 3.91 | 3.22-4.64 | 2.56 | 1.99-3.23 |
| 6-12 months | **3.88** | **3.25-4.52** | **2.59** | **2.09-3.13** |
| 1-2 years | 3.17 | 2.70-3.69 | 2.79 | 2.40-3.20 |
| 2-5 years | **3.65** | **3.15-4.26** | **2.86** | **2.52-3.22** |

Statistically significant difference (P < 0.05) in bold.

Supplementary Table S3: Mean contact frequency per period, calculated as mean contact frequency per year for each period, for former ICU patients and reference patients with 95% Confidence Interval (CI) as shown in Figure 3

| Period | Former ICU patients (mean) | 95% CI | Reference patients  (mean) | 95% CI |
| --- | --- | --- | --- | --- |
| -12-0 months | **19.61** | **17.31-22.17** | **10.02** | **7.81-12.38** |
| 0-3 months | **39.20** | **32.48-47.55** | **8.87** | **7.27-10.52** |
| 3-6 months | **20.26** | **17.25-23.34** | **9.83** | **7.89-12.16** |
| 6-12 months | **20.20** | **17.19-23.42** | **10.28** | **8.69-11.88** |
| 1-2 years | **22.60** | **17.48-29.23** | **11.48** | **9.84-13.21** |
| 2-5 years | **18.53** | **15.58-21.85** | **12.03** | **10.33-13.91** |

Statistically significant difference (P < 0.05) in bold.

Supplementary Table S4. Rate Ratio (95%CI) of ICU (N = 199) and reference (N = 199) cohort respectively for developing a new disease-episode in each post-ICU timeframe per ICPC-2 chapter compared to one year before ICU admission.

| Disease-episode ICPC-2 category | 0-3 months  ICU;Reference | 3-6 months  ICU;Reference | 6-12 months  ICU;Reference | 1-2 years  ICU;Reference | 2-5 years;  ICU;Reference |
| --- | --- | --- | --- | --- | --- |
| *A* | **3.59 (2.75-4.67); 2.24 (1.63-3.06)** | **2.319 (1.73-3.11); 2.62 (1.92-3.56)** | **1.72 (1.31-2.26);** 1.28 (0.94-1.75) | 0.94 (0.72-1.23);  0.95 (0.71-1.27) | 0.99 (0.78-1.26);  0.74 (0.57-0.97) |
| *B* | **3.30 (2,28-4.78); 3.31 (2.22-4.94)** | **3.23 (2.22-4.71); 3.31 (2,21-4,97)** | **1.65 (1.13-2.41);**  **1.98 (1.34-2.94)** | 0.81 (0.55-1.19); 1.00 (0.67-1.48) | **0.39 (0.27-0.57); 0.40 (0.27-0.60)** |
| *D* | **2.70 (1.90-3.84);**  **2.93 (2.01-4.27)** | **2.58 (1.80-3.69); 3.28 (2.26-4.77)** | **1.71 (1.21-2.41); 1.78 (1.23-2.58)** | 1.02 (0.72-1.43);  1.04 (0.72-1.50) | **0.48 (0.34-0.67);**  **0.63 (0.44-0.89)** |
| *F* | **2.71 (1.90-3.87); 2.91 (2.03-4.17)** | **3.06 (2.15-4.35); 2.85 (1.97-4.11)** | **1.65 (1.16-2.34);**  **1.74 (1.22-2.48)** | 0.85 (0.60-1,21);  0.95 (0.67-1.35) | **0.41 (0.29-0.57); 0.42 (0.30-0.60)** |
| *H* | **3.17 (2.07-4.84); 3.60 (2.39-5.41)** | **3.17 (2.07-4.87); 2.88 (1.88-4.41)** | **1.77 (1.16-2.72); 2.18 (1.45-3.27)** | 1.10 (0.73-1.67); 1.05 (0.69-1.58) | **0.56 (0.37-0.85); 0.65 (0.43-0.96)** |
| *K* | **2.42 (1.78-3.30); 2.78 (2.03-3.79)** | **1.91 (1.37-2.66); 2.12 (1.52-2.97)** | 1.14 (0.83-1.56); 1.27 (0.91-1.75) | 0.66 (0.48-0.91); 0.71 (0.51-0.97) | **0.31 (0.23-0.42); 0.36 (0.26-0.48)** |
| *L* | 0.90 (0.55-1.49); 0.65 (0.35-1.19) | 0.99 (0.61-1.61); 1.01 (0.60-1.69) | 0.89 (0.60-1.33); 1.06 (0.71-1.58) | 0.92 (0.67-1.26); 1.05 (0.76-1.46) | 1.12 (0.85-1.44); 0.99 (0.75-1.31) |
| *N* | **5.09 (3.56-7.26); 3.54 (2.40-5.20)** | **3.34 (2.27-4.91); 3.50 (2.37-5.17)** | **1.89 (1.29-2.77); 1.80 (1.22-2.67)** | 1.08 (0.74-1.57); 1.19 (0.81-1.73) | **0.53 (0.37-0.76); 0.45 (0.31-0.66)** |
| *P* | **3.27 (2.17-4.93); 2.62 (1.74-3.94)** | **3.65 (0.43-5.48); 2.60 (1.72-3.92)** | **1.92 (1.28-2.89); 1.61 (1.08-2.41)** | 0.93 (0.62-1.41); 0.86 (0.58-1.29) | **0.53 (0.35-0.79); 0.34 (0.23-0.51)** |
| *R* | **2.86 (2.018-4.06); 3.48 (2.42-4.99)** | **2.46 (1.71-3.54); 3.00 (2.06-4.37)** | **1.57 (1.10-2.22); 1.82 (1.26-2.62)** | 0.99 (0.71-1.40); 1.17 (0.82-1.67) | **0.62 (0.45-0.85);** 0.72 (0.51-1.00) |
| *S* | **2.47 (1.91-3.20); 2.24 (1.69-2.97)** | **3.30 (2.60-4.20); 4.35 (3.43-5.51)** | **1.60 (1.25-2.05); 1.90 (1.48-2.43)** | 0.96 (0.76-1.22); 0.94 (0.67-1.33) | **0.75 (0.61-0.91);** 1.04 (0.77-1.40) |
| *T* | **3.32 (2.43-4.54); 3.11 (2.27-4.26)** | **3.33 (2.42-4.57); 3.11 (2.27-2.26)** | **1.69 (1.23-2.33); 1.58 (1.15-2.17)** | 0.91 (0.66-1.25); 0.82 (0.60-1.13) | **0.41 (0.30-0.55); 0.33 (0.24-0.45)** |
| *U* | **3.81 (2.35-6.15); 3.16 (1.92-5.19)** | **2.38 (1.43-3.95); 2.65 (1.59-4.41)** | **1.68 (1.03-2.76);** 1.36 (0.81-2.27) | 1.01 (0.62-1.66); 1.05 (0.64-1.73) | **0.61 (0.37-0.99);** 0.62 (0.38-1.02) |
| *W* | **2.02 (1.42-2.85); 1.95 (1.32-2.86)** | **1.96 (1.38-2.80); 2.59 (1.80-3.74)** | 0.98 (0.68-1.40); 1.31 (0.91-1.90) | **0.50 (0.45-0.71);** 1.18 (0.85-1.65) | **0.33 (0.24-0.45); 0.67 (0.49-0.93)** |
| *X* | **3.79 (2.65-5.42); 3.42 (2.35-4.98)** | **3.38 (2.34-4.90); 3.42 (2.34-5.01)** | **1.58 (1.08-2.30); 2.08 (1.44-3.02)** | 0.95 (0.66-1.37); 1.02 (0.70-1.48) | **0.41 (0.29-0.60); 0.53 (0.37-0.77)** |
| *Y* | **3.40 (1.61-7.18); 3.08 (1.53-6.22)** | **2.98 (1.36-6.55); 2.74 (1.32-5.67)** | **2.27 (1.12-4.60);** 1,23 (0,58-2,61) | 0.84 (0.39-1.80); 0.76 (0.37-1.56) | **0.42 (0.21-0.84); 0.41 (0.22-0.78)** |
| *Z* | **3.21 (2.33-4,43); 2.90 (2.11-3.99)** | **3.13 (2.26-4.35); 2.87 (2.08-3.97)** | **1.59 (1.14-2.21); 1.44 (1.04-2.00)** | 0.85 (0.61-1.18); 0.76 (0.55-1.05) | **0.35 (0.25-0.48); 0.35 (0.25-0.47)** |

Statistically significant difference (P < 0.05) in bold.

Supplementary Table S5. Rate of two Rate Ratios (95%CI) for developing a new disease-episode in each post-ICU timeframe per ICPC-2 chapter compared to reference cohort and one year before ICU admission.

| Disease-episode ICPC-2 category | 0-3 months | 3-6 months | 6-12 months | 1-2 years | 2-5 years |
| --- | --- | --- | --- | --- | --- |
| *A* | **1.60 (1.06-2.42)** | 0.89 (0.58-1.36) | 1.34 (0.89-2.03) | 0.99 (0.67-1.48) | 1.33 (0.93-1.91) |
| *B* | 1.00 (0.58-1.72) | 0.98 (0.56-1.70) | 0.84 (0.48-1.44) | 0.81 (0.47-1.41) | 0.98 (0.57-1.69) |
| *D* | 0.92 (0.55-1.55) | 0.79 (0.47-1.32) | 0.96 (0.58-1.59) | 0.97 (0.59-1.61) | 0.76 (0.47-1.24) |
| *F* | 0.93 (0.56-1.55) | 1.07 (0.65-1.79) | 0.95 (0.57-1.58) | 0.90 (0.55-1.48) | 0.96 (0.59-1.56) |
| *H* | 0.88 (0.49-1.59) | 1.10 (0.60-2.02) | 0.81 (0.45-1.47) | 1.05 (0.58-1.89) | 0.88 (0.49-1.55) |
| *K* | 0.87 (0.56-1.35) | 0.90 (0.56-1.44) | 0.90 (0.57-1.42) | 0.94 (0.60-1.47) | 0.86 (0.57-1.35) |
| *L* | 1.39 (0.63-3.06) | 0.98 (0.48-2.00) | 0.84 (0.48-1.47) | 0.87 (0.55-1.38) | 1.12 (0.77-1.64) |
| *N* | 1.44 (0.85-2.43) | 0.95 (0.55-1.65) | 1.05 (0.61-1.82) | 0.91 (0.53-1.55) | 1.17 (0.69-1.99) |
| *P* | 1.25 (0.70-2.23) | 1.41 (0.79-2.51) | 1.20 (0.67-2.12) | 1.08 (0.61-1.93) | 1.56 (0.88-2.76) |
| *R* | 0.82 (0.50-1.36) | 0.82 (0.49-1.39) | 0.86 (0.52-1.43) | 0.85 (0.52-1.39) | 0.86 (0.54-1.37) |
| *S* | 1.11 (0.75-1.62) | 0.76 (0.54-1.07) | 0.85 (0.60 (1.20) | 0.94 (0.67-1.33) | 1.04 (0.78-1.33) |
| *T* | 1.07 (0.69-1.66) | 1.07 (0.68-1.67) | 1.07 (0.68-1.68) | 1.11 (0.71-1.73) | 1.25 (0.81-1.93) |
| *U* | 1.21 (0.61-2.41) | 0.90 (0.44-1.85) | 1.24 (0.61-2.53) | 0.96 (0.47-1.95) | 0.97 (0.48-1.95) |
| *W* | 1.04 (0.62-1.74) | 0.76 (0.46-1.26) | 0.75 (0.45-1.25) | **0.42 (0.26-0.69)** | **0.49 (0.31-0.77)** |
| *X* | 1.11 (0.66-1.86) | 0.99 (0.58-1.68) | 0.76 (0.45-1.29) | 0.94 (0.55-1.59) | 0.77 (0.46-1.29) |
| *Y* | 1.10 (0.40-3.08) | 1.09 (0.37-3.18) | 1.84 (0.66-5.16) | 1.10 (0.39-3.12) | 1.01 (0.39-2.60) |
| *Z* | 1.11 (0.70-1.74) | 1.09 (0.69-1.73) | 1.10 (0.69-1.75) | 1.12 (0.70-1.77) | 1.01 (0.64-1.58) |

Statistically significant difference (P < 0.05) in bold.
